# Cadmium exposure and risk of breast cancer: A meta-analysis

**DOI:** 10.1016/j.envres.2022.115109

**Published:** 2022-12-20

**Authors:** VA. Florez-Garcia, EC. Guevara-Romero, MM. Hawkins, LE. Bautista, TE. Jenson, J. Yu, AE. Kalkbrenner

**Affiliations:** a Joseph J. Zilber School of Public Health, University of Wisconsin-Milwaukee, Milwaukee, WI, 5321, USA; b Department of Public Health. Universidad Del Norte, Barranquilla. Colombia; c Public Health. Carroll University College of Health Sciences. 100 N East Ave, Waukesha, WI, 53186, USA; d Department of Population Health Sciences, School of Medicine and Public Health, University of Wisconsin, 610 Walnut Street, WARF 703, Madison, WI, 53726-2397, USA

**Keywords:** Cadmium, Heavy metals, Breast cancer, Environmental exposure, Epidemiology

## Abstract

**Background:**

Cadmium is a heavy metal with carcinogenic properties, highly prevalent in industrialized areas worldwide. Prior reviews evaluating whether cadmium influences breast cancer have been inconclusive and not reflected several recent studies.

**Objective:**

To evaluate the association between cadmium exposure and female breast cancer incidence, with an emphasis on separately estimating dietary vs. airborne vs. biomarker measures of cadmium and studies published until October 2022.

**Methods:**

We evaluated risk of bias using set criteria and excluded one study judged to have high risk based on self-report of breast cancer and insufficient adjustment. We conducted a random effects meta-analysis of epidemiological studies, including subgroups by exposure route and by menopausal status.

**Results:**

A total of 17 studies were eligible for our meta-analysis. Only 2 studies addressed airborne cadmium directly. Breast cancer risk was elevated in women exposed to higher levels of cadmium across all studies − pooled odds ratio: 1.13 (95% confidence interval: 1.00, 1.28), with notable heterogeneity between studies (*I*^2^_=_ 77%). When examining separately by exposure route, dietary cadmium was not linked with an elevated risk – (OR: 1.05; 95%CI: 0.91, 1.21; *I*^2^ = 69%), consistent with prior reviews, but biomarker-based studies showed an elevated but non-significant pooled measure (OR: 1.37; 95%CI: 0.96, 1.94; *I*^2^ = 84%). We did not observe any clear patterns of different risk by menopausal status.

**Conclusion:**

Findings from our meta-analysis suggest that exposure to higher cadmium increases the risk of breast cancer in women, but with remaining questions about whether non-dietary exposure may be more risky or whether residual confounding by constituents of tobacco smoke may be at play.

## 1. Introduction

Breast cancer (BC) is the main cause of cancer deaths among women in both high- and middle-income countries (IARC, 2022), constituting a world-wide health concern. INC, 2020, there were about 2.26 million new cases from female BC worldwide (IARC, 2022; Sung et al., 2021). Indeed, according to the American Cancer Society, BC is the most common type of cancer among women in the United States, with 287, 850 new cases of in 2022 (Siegel et al., 2022). Likewise, this is the most common cancer in Europe, with 531,000 new cases INC, 2020 (IARC, 2022).

Breast cancer is a worldwide public health concern. BC incidence patterns in low- and middle-income countries may be lower than those in high-income countries (age-standardized rate [ASR] 29.7 versus 55.9 per 100,000, respectively) (Arnold et al., 2022). However, BC statistics for these transitioning countries are subject to misclassification and are often rough estimates, as national cancer registries with cancer data collection often do not exist. BC is the most common cancer among Colombian women, with an incidence rate of 44.1 per 100,000. (Florez-Lozano et al., 2019; IARC, 2018; INC, 2020). Similarly, in Peru, Chile, and Ecuador, BC is the most frequent cancer among women (IARC, 2018).

In all scenarios, women living in urban and densely populated areas have the highest risk of BC (Fei et al., 2015; Florez-Lozano et al., 2019; Solikhah et al., 2019), which suggests the existence of environmental causes. Exposure to heavy metals, which is likely higher in urban areas, is associated with several types of cancer, including BC (Adams et al., 2016; Caffo et al., 2015; Wu et al., 2019). Currently, cadmium is the most-studied heavy metal exposure in epidemiological studies of BC; hence, we have restricted our review to cadmium exposure from all sources. Cadmium has been classified as a carcinogen and risk factor in lung cancer (Nawrot et al., 2006), and its intake has been pointed out as related to cancer in general.(Cho et al., 2013). Moreover, cadmium can bind to estrogen receptors alpha through an interaction with the hormone-binding domain of the receptor (Stoica et al., 2000), and is involved in the inhibition of DNA repair (Schwerdtle et al., 2010). Both molecular mechanisms are important in BC carcinogenesis.

Several prior reviews and meta-analyses have been conducted about cadmium and BC incidence (Cho et al., 2013; Larsson et al., 2015; Lin et al., 2016; Van Maele-Fabry et al., 2016). Taken together, these reviews do not support one clear conclusion regarding whether cadmium exposure is or is not causally implicated in BC. Several mention heterogeneity in findings and the need to separately examine by subgroups. Four of the reviews focused only on the dietary route of cadmium exposure, and these 4 all found near-null and imprecise summary measures of association (Cho et al., 2013; Lin et al., 2016; Van Maele-Fabry et al., 2016). In contrast, a review that emphasized biomarker exposure measures integrating all routes of exposure reported a notable and significant summary measure of 2.24 (95% CI 1.50, 3.34) for the highest vs. lowest category of cadmium (Larsson et al., 2015). These discrepancies point out the potential importance of route of exposure as an important feature of the potential cadmium-BC link.

The importance of directly examining airborne cadmium exposure distinct from ingested cadmium is supported by several observations. Airborne cadmium may result in higher effective doses; once cadmium is in the lungs, from 10% to 50% of an inhaled dose gets into the bronchoalveolar barrier reaching out to the bloodstream (HHS, 2011). In contrast, gastrointestinal absorption of the more common dietary route of cadmium is only about 6% and may be influenced by nutritional factors, such as iron status (Jarup, 2003), with possible differences in gastrointestinal absorption of cadmium in water vs. food (EPA-IRIS, 2006). Thus, air cadmium can readily go into the body, and activate estrogen receptor-α, induce the proliferation of estrogen-dependent BC cells, and increase the expression of estrogen-regulated genes (Martin et al., 2003; Siewit et al., 2010). Another argument for the importance of airborne cadmium comes from a recent study finding that it was related to the more aggressive estrogen/progesterone receptor-negative BC when comparing among women with BC (case-only study) (Kresovich et al., 2019). A factor (like airborne cadmium) related to BC subtype may have a greater likelihood of playing an etiologic role for BC incidence.

Given that cadmium is a modifiable toxic exposure with an as-yet unresolved role on BC risk, we performed a systematic review and meta-analyses. We especially focused on subgroup analyses by route of exposure (e.g. airborne vs. diet vs. biomarker) and also examined another subgroup where sufficient numbers of studies allowed this: menopausal status. Furthermore, we included several studies published since the most recent review INC, 2020, to provide the most comprehensive perspective on the state of the evidence.

## 2. Methodology

We followed the Preferred Reporting Items for Systematic Review and Meta-analysis Guidelines − PRISMA− guidelines to prepare this review (Moher et al., 2015).

### 2.1. PECO statement and eligibility criteria

We define our Population, Exposure, Comparator, and Outcomes (PECO) as follows:

**Population**: Female adults (age ≥18 years)

**Exposure:** Cadmium (from any source) measured by biomarker, diet, or air measurement.

**Comparator:** Lower or higher levels of cadmium.

**Outcomes**: Incident cases of breast cancer.

**Study designs**: We included case-control and cohort study designs. We excluded cell models, ecological studies, case reports, and descriptive studies that lacked a measure of association between cadmium and breast cancer.

**Dates and language:** All studies published until October 2022 were included. Searches were conducted in English, but articles in English and Spanish were included in the assessment. In studies that utilized the same population and the same medium for measuring the exposure, we chose the most recent published article in order to avoid duplicating the population in the meta-analysis.

### 2.2. Search strategy

We searched for publications using PubMed (MEDLINE), SCOPUS, and Web of Sciences, using the keywords: heavy metal and breast cancer. The initial search was crafted in PubMed using a combination of Medical Subject Heading (MeSH) terms to guide the vocabulary. MeSH matches a variety of keywords without the use of synonyms in MEDLINE and CENTRAL searches, and provides a comprehensive solid foundation for the search process and transferred well to other platforms. The same search strategy was conducted in the other databases. The search contained the following terms 1) Heavy metal prioritized and related (“cadmium” OR “lead” OR “mercury” OR “chromium” OR “arsenic” OR “metallic air pollutants” OR “heavy metal”). 2) Breast cancer “breast cancer” OR “breast neoplasms” OR “breast tumor”) and 3) exclusions for irrelevant factors and factors outside the scope of this review (NOT “review” OR “case report” OR “mice” OR “mouse” OR “in vitro”). We broadly searched across all metals to capture papers that reported cadmium results within a suite of metals examined. Only studies in humans were included. To increase the sensitivity of the search, we conducted a PubMed search without the above-mentioned exclusions. Searches were conducted throughout February and March 2021 and then updated in October 2022. (Supplemental Tables S–1: Search strategy used in the review).

### 2.3. Study selection and data extraction

Two reviewers (VFG & EGR) independently screened retrieved articles for eligibility and extracted the following data from elegible studies using a pilot-tested form: the first author, publication year, design, location, duration of follow-up (cohort studies identified), exposure of interest (cadmium), exposure measurement (biomarker, air sample, etc.), subject’s age, menopausal status (pre, post, & overall) recruitment period, relative risk point estimate and 95% CIs from the fully adjusted model, cut-off values for each category of exposure, sample size, and variables adjusted for in the multivariate analysis.

### 2.4. Risk of bias in individual studies

To assess the potential for systematic errors in each study we used the preliminary Risk of Bias (RoB) in Non-randomized Studies of Exposures (ROBINS-E) tool (Morgan et al., 2019), which includes bias due to: (a) confounding; (b) selection of participants into the study; (c) exposure misclassification; (d) missing data; (e) outcome measurement error; and (f) selective reporting of results (Supplemental Table 2). For each domain we scored the risk of bias as low, moderate, or high, and assigned the lowest score to the whole article. For example, if at least one domain had a moderate risk of bias, then the whole article was categorized as having moderate bias. Any disagreement was resolved by discussion and consensus by the two scorers (VFG and EGR).

### 2.5. Synthesis of results

We conducted a narrative synthesis of data extracted from the studies. Also, we performed a quantitative analysis based on categorical exposure to cadmium. The original measures of association (e.g., odds ratio –OR, risk ratio –RR, etc.), and the inverse of their variances were explored in a forest chart to compare the results while keeping the original confidence intervals (95%CI).

We pooled the effect estimates from each study, independently of the study design, using a random-effects model. Because we were interested in generalizing beyond these included studies, we were waiting for some heterogeneity and we were conservative in the interpretations, we preferred the random effects model, although we also checked whether results differed for the overall meta-analysis when using a fixed effect model (Tufanaru et al., 2015; Higgins et al., 2022). We conducted analyses separately by menopausal status (pre, post, and both) since current knowledge suggests a key role of age and the hormonal mileau in the pathogenesis of BC. Additionally, we conducted analysis by type of exposure measurement used in the study: cadmium in air, dietary cadmium and cadmium body burden measured with a biomarker. We estimated the pooled overall effect for all subjects. The relative risk for the highest exposure category compared to the lowest one was used in all cases. The *I*^2^ statistic was used to assess the level of heterogeneity of the effect across studies.

We used meta-regression to evaluate the extent to which study designs (case-control vs. cohort) contributed to the heterogeneity of effects across studies. In addition, we used funnel plots to assess potential publication bias. The analysis was done with RevMan (Review Manager, version 5.4, 2014, The Nordic Cochrane Center, The Cochrane Collaboration, Copenhagen, Denmark) and Stata 14. The protocol for this study is available in PROSPERO (http://www.crd.york.ac.uk/PROSPERO/; CRD42022341929).

## 3. Results

**Fig. 1.**
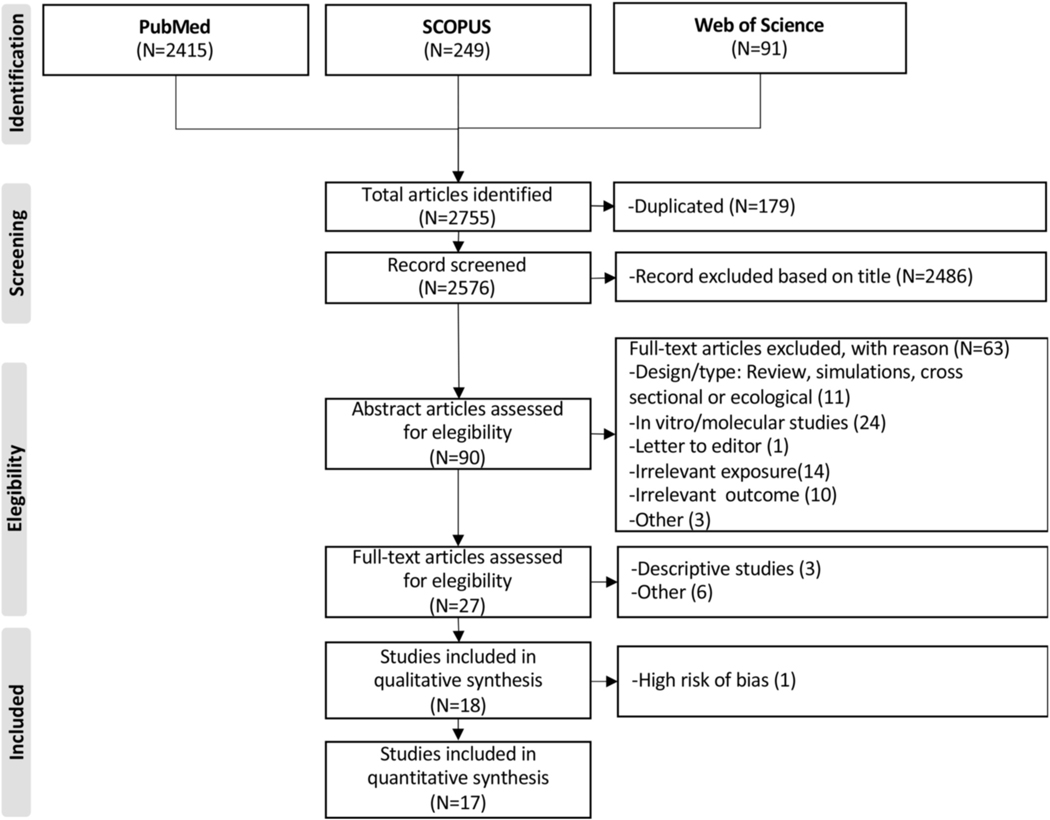
Prisma flow-chart of systematic literature search on cadmium exposure and breast cancer.

We found 2755 articles potentially eligible for our study. After screening titles and duplicated studies, 2486 articles were excluded. Thus, the abstract of 90 articles were screened for eligibility. From these, 72 were excluded because they did not allow for the estimation of the effect of cadmium on BC, such as ecological or in vitro studies. A total of, 18 articles were included in this review, and 17 were included in the meta-analysis (Fig. 1).

A case-control design was used in 50% (9/18) and a cohort design in 50% (9/18) of the articles (Table 1). Studies were conducted in the United States (7/18), Europe (7/18), Japan (3/18), and China (1/18). Menopausal status was not reported in four studies (Gaudet et al., 2019; McElroy et al., 2006; Nagata et al., 2013; Sawada et al., 2012).

### 3.1. Assessment of risk of bias

Among all studies, (17/18) had a moderate risk of bias (Supplemental Tables S2 and S3). In most cases, this was due lack of accuracy in the measurement of the outcome or lack of adjustment for known confounding factors. Only one study (Gallagher et al., 2010) was classified as having a high risk of bias, because there was not adjustment for body mass index and the outcome (BC) was self-report. Results of this study were excluded from the pooled analysis to preserve the validity of the overall estimate. Funnel plots revealed no evidence of publication bias

### 3.2. The cadmium-breast cancer association

**Fig. 3.**
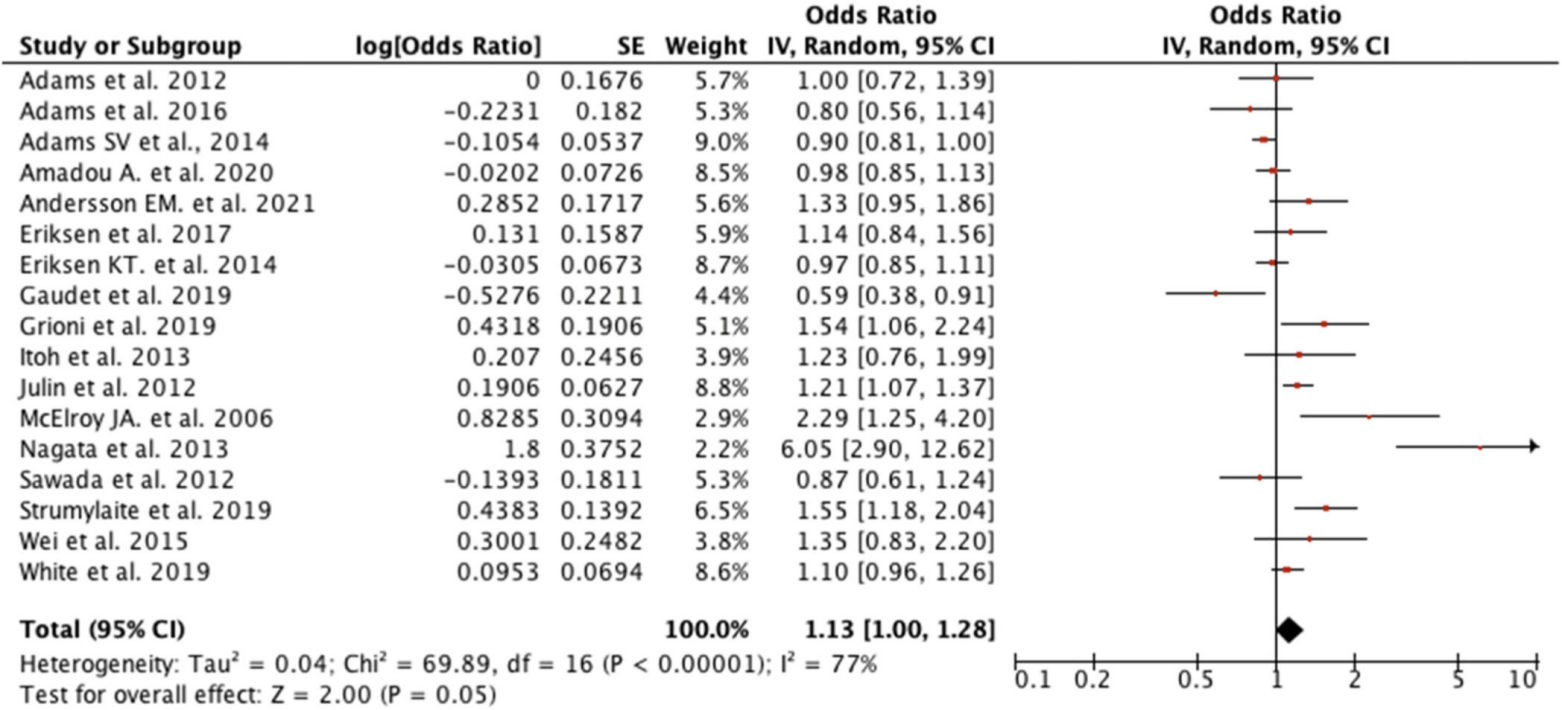
Forest plot for overall women.

Our overall pooled estimate for 17 studies showed that women with higher cadmium exposure were 1.13 times more likely to develop BC (OR: 1.13; 95%CI: 1.00, 1.28), and there was considerable heterogeneity across studies (*I*^2^ = 77%) (Fig. 3). Despite evidence for heterogeneity, there was little difference between random and fixed effects estimates, and so for this and our other pooled estimates, we report on the random effects estimate. Four studies (Gaudet et al., 2019; Julin et al., 2012; Nagata et al., 2013; Strumylaite et al., 2019) contributed to most of the heterogeneity. After excluding those studies, summary measure was slightly attenuated and became in statistically non-significant (OR: 1.06; 95%CI:0.96, 1.17; *I*^2^:54%).

**Fig. 4.**
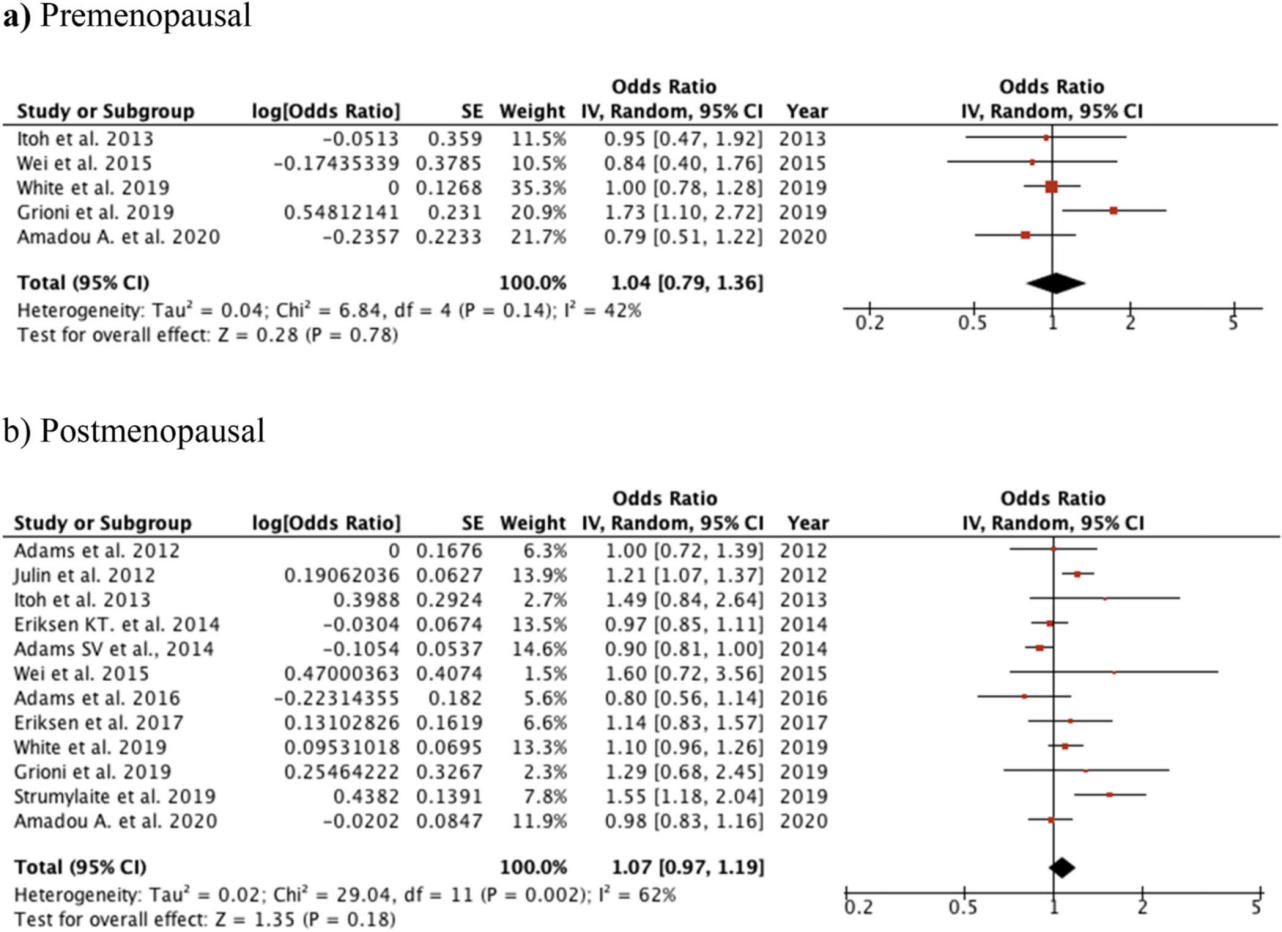
Forest plot of subgroups according to menopausal status.

Among premenopausal women (4 studies), there was a slightly elevated and non-statistically significant association between cadmium and BC (OR: 1.04; 95%CI: 0.79, 1.36; *I*^2^: 42%) (Fig. 4a). Similar results were found in a group of 12 studies on postmenopausal (OR: 1.07; 95% CI: 0.97, 1.19; *I*^2^ = 62%). with little difference between fixed and random effects polled estimates (Fig. 4b). Despite the difference in the frequency of cadmium exposure in case-control and cohort studies, a meta–regression analysis showed that study design was not a significant source of heterogeneity (*P*_Het_ = 0.190).

Additional subgroup analysis based only on the 8 studies that measured cadmium body burden with biomarkers (blood and urinary cadmium) resulted in a meaningfully-elevated summary measure with BC risk that was not statistically (OR: 1.37; 95%CI: 0.96, 1.94; *I*^2^ = 84%). Similar results were found when limiting to the 4 studies of biomarker exposures (OR:1.19; 95%CI: 0.86, 1.65; *I*^2^ = 66%) (Supplementary Fig. S1). In studies measuring dietary cadmium (Supplementary Fig. S2), the risk of BC in exposed women was similar as non-exposed women (OR: 1.05; 95%CI: 0.91, 1.21; *I*^2^ = 69%). with similar patterns for postmenopausal women and a highly imprecise summary measure for 2 results for premenopausal women. Finally, our findings did not support the hypothesis that airborne cadmium exposure plays a role in BC yielding a summary OR (from 2 studies) of 1.04; 95% CI: 0.93, 1.16), with similar results after stratifying by menopausal status (Supplemental Fig. S3).

## 4. Discussion

When pooling across 17 studies of cadmium exposure (measured in various ways) and BC incidence. We found a positive association reflecting 13% in the risk of BC. The size of this increased risk with higher cadmium is strikingly similar to that of the most recent meta-analysis by Filippini. (Filippini et al., 2020): 12% increased risk, although the Filipini review, which included fewer studies (8) of BC incidence, did not find a statistically-resolved effect.

Our study strengths include the evaluation of different sources of exposure and exposure measurements, the inclusion of multicentric studies and studies with different designs. Due to the scarcity of high quality data on the cadmium-BC relationship, these strategies allowed a more comprehensive evaluation of the issue. Further strengths of our review include that it is more comprehensive and updated – including a larger number of studies. We had a clear definition of our outcome – focusing on BC incidence (excluding studies on BC mortality). It is widely accepted that risk factors for mortality may differ from risk factors associated with disease incidence. Indeed, as recognized by the authors of prior reviews, blending BC incidence and mortality endpoints could have been an important source of heterogeneity in their studies. Importantly, we separately examined cadmium by route of exposure/ measurement type, including the first meta-analysis of airborne cadmium and BC. This also helped to shed light on possible sources of bias and helped to integrate with prior reviews, some of which only focused on dietary cadmium.

Our subgroup analyses focusing on discrete sources of exposure - dietary and airborne cadmium - did not show an increase in risk, with near-null pooled estimates for both. Previous reviews of the dietary route of cadmium similarly found near-null estimates (Lin et al., 2016; Van Maele-Fabry et al., 2016), constituting an increasing body of evidence that dietary cadmium may not increase BC risk. While we hypothesized that airborne cadmium may constitute a unique and more risky exposure, our analysis did not bear this out. Yet at this time only 2 studies have focused on airborne cadmium and BC risk (Amadou et al., 2020; White et al., 2019), limiting the robustness of any conclusions regarding a role for airborne cadmium.

In contrast, our summary measure of biomarker studies – which reflect all sources of exposure - revealed an elevated association, a notable 37% increase in BC risk, and while this result did not reach statistical significance, this was influenced by the limited number of studies (8) versus the 17 included in the overall estimate. The magnitude of this summary OR is consistent with results of prior reviews including those by Larsson et al., (2015) and Lin et al., (2016)(Larsson et al., 2015; Lin et al., 2016), but not other prior reviews (Filippini et al., 2020). Of all the measures of cadmium exposure, only the biomarkers reflect exposure from tobacco smoke, which is an important source of cadmium exposure at the population-level (Menke et al., 2009). It is because of the importance of tobacco as a source of cadmium exposure that we considered it an essential adjustment variable in our risk of bias criteria. Yet adjusting for smoking status will not fully eliminate the possibility that a cadmium biomarker among smokers may be serving as a proxy for other constituents of tobacco smoke that may be cancer-promoting. It is possible that the existing cadmium-BC literature has not yet fully addressed this possibility: that cadmium BC measures of association may suffer from residual confounding by constituents of tobacco smoke rather than reflecting a causal influence of cadmium itself. An alternate explanation for the high (albeit non-significant) pooled measure of association between cadmium biomarkers and BC is that some biomarkers are a superior measure of cadmium reflecting long past periods of exposure that are etiologically relevanat for BC risk.

Menopausal status could delineate different responses to cadmium exposure and BC risk. While we separately pooled results for pre-menopausal and post-menopausal status, also within exposure route, the results were impacted by increasingly-smaller numbers of included studies and limited statistical precision. No clear pattern of increased susceptibility by menopausal status was evident.

This multi-source meta-analysis included articles with a moderate risk of bias in outcome measurement. Nevertheless, they were conducted in high-income countries, and most were based in cancer registries. Therefore, our findings may not be applicable to population in low and middle-income countries, where cadmium exposure levels may be substantially different.

An unavoidable limitation, arising in part from the nature of studying rare endpoints like BC, is the lack of data on exposure accrual time and induction period. BC may develop based on exposures over many previous decades. Each individual study included differed in the latency period reflected based on the timing of questionnaires or whether a biomarker was from urine or blood. For example, cadmium is slowly excreted in urine resulting in accumulation in body tissues, and a biological half-life up to 30 years (Van Maele-Fabry et al., 2016). We were unable to separately examine by timing of questionnaire or specific type of biomarker due to a limited number of studies available for these subgroups. Future studies, based on meta-analysis of individual data could shed light on source-related heterogeneity of the cadmium-BC relationship. Similarly, while estrogen receptor subtype is an important feature of BC and could delineate subgroups with differing response to cadmium exposure. The lack of data in the original studies curtailed the assessment of the role of estrogen receptors as a source of heterogeneity in the effect of cadmium on BC. An important research issue, outside the scope of our study, but of great importance, is the relationship between cadmium and BC cancer progression and BC mortality. From a public health perspective, we encourage tackling these knowledge gaps in future studies, both in high- and low-middle-income populations.

In conclusion, our results add evidence for a role of cadmium exposure in BC with some heterogeneity, but with some caveats based on the possibility of residual confounding by constituents of tobacco smoke, which could be themselves cancer-promoting exposures driving observed associations with cadmium biomarkers.

## Supplementary Material

Multimedia component 1

## Figures and Tables

**Fig. 2.**
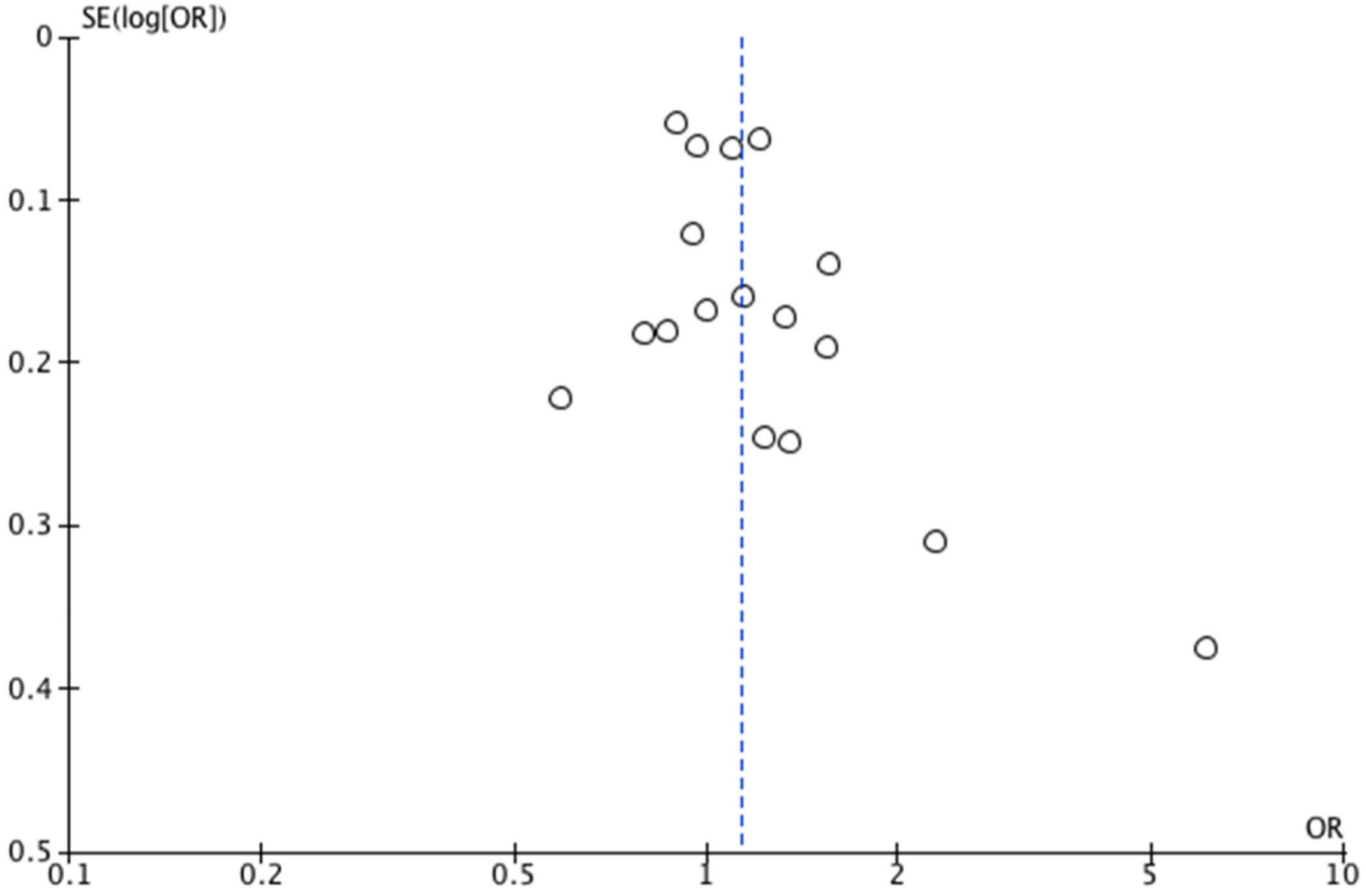
Funnel plot of comparison: Overall female breast cancer.

**Table 1:** Characteristics of the studies included in the systematic review.

First author	Design	Location	Exposure Measurement	Type of Population	Number of cases	Sample size	Measures of Central Tendency & Dispersion	Adjustment Variables^a^

Andersson et al., (2021)	Case-Control	Sweden	Biomarker (BCL)	Overall	1274	3846	Highest (1.20–4.997) vs. Lowest (0.041–0.409 quartile (μg/L)	Parity, age at first child, late menopause, HRT, BMI, alcohol, physical activity, smoking, fiber consumption, socioeconomic index
Amadou et al., (2020)	Case-Control	France	Air	Premenopausal Postmenopausal Overall	4059	8118	Highest (>5.47) vs Lowest (≤0.033) quintile (mg/m^2^)	Physical activity, smoking status, level of education, BMI, previous family history of breast cancer, history of personal benign breast disease, age at first full-term pregnancy, parity, breastfeeding, oral contraceptive use, menopausal HRT use and status of birthplace.
White et al., (2019)	Cohort	US	Air	Premenopausa Postmenopausal Overall	2587	50,884	No available. However, authors compared highest with lowest Cd quintiles (μg/m^3^)	Race, education, annual household income, marital status, parity, census-track level median income and geographic region.
Gaudet et al., (2019)	Case- Control	US, Italy, Sweden	Biomarker (BCL)	Overall	1435	2868	Highest (>2.00) vs Lowest (0–0.49) quintiles (μg/L)	Race, blood draw date, age, cigarette smoking initiation relative to first birth, cigarette smoking status, iron/ multi-vitamin use, alcohol consumption, HRT, and season of blood draw
Grioni et al., (2019)	Cohort	Italy	Diet	Premenopausal Postmenopausal Overall	451	8924	Highest (8.82–16.10) vs Lowest (0.45–6.72) quintile (μg/day)	Age, energy intake, menopausal status, age at menarche, height, BMI, age at first childbirth, smoking status, years of education, alcohol, vegetable intake, dietary iron, dietary calcium and dietary zinc
Strumylaite et al., (2019)	Case-Control	Lithuania	Biomarker (UCL)	Postmenopausal	509	1679	Highest (>0.33) vs lowest (<0.18) tertil of creatinine-adjusted urinary cadmium (kg 10^−9^/kg 10^−3^ creatinine)	Age, number of births, age at first birth, estrogen-active (fertile) period, HRT during menopause, family history on breast cancer, alcohol use, smoking, BMI, education, marital status, diabetes mellitus, and thyroid diseases
Adams et al., (2016)	Case-cohort	US	Biomarker (UCL)	Postmenopausal	508	12,701	Highest (>0.748) vs Lowest (< 0.325) quartile (μg/g-Creatinine)	Age group and adjusted for Women’s Health Initiative (WHI) study component, age at first birth, age at menopause, family history of breast cancer, smoking status, pack-years of smoking, body mass index, education, alcohol consumption, WHI Hormone Therapy Trial arm, and hormone therapy use.
Eriksen et al., (2017)	Cohort	Denmark	Biomarker (UCL)	Postmenopausal	900	1978	Highest (0.42–5.41) vs Lowest (<0.16) tertile (ng/ mL)	Educational level, number of births, age at first birth, HRT status, HRT use, height, weight, physical activity and alcohol intake
Wei et al., (2015)	Case-Control	China	Biomarker (UCL)	Premenopausal Postmenopausal Overall	240	486	Highest (>2.36) vs. Lowest (1.59) tertile (μg/g creatinine)	Age, BMI, age at menarche, marital status, education, parity, menopausal status, and family history of breast cancer
Adams et al., (2014)	Cohort	US	Diet	Postmenopausal	6658	150,889	Highest (>14.21) vs. Lowest (<7.10) quintile (μg/day)	Total energy intake, age and study component, BMI, smoking, alcohol, consumption, race/ethnicity, education, physical activity, age at first birth, age at menarche, age at menopause, unopposed estrogen use, and estrogen and progesterone use, mammography 2 years before baseline, daily vegetable servings and daily grain servings.
Eriksen et al., (2014)	Cohort	France	Diet	Postmenopausal	1390	23,815	Highest (>0.15) vs. Lowest (<11.9) tertiles (μg/day)	Educational level, smoking status, number of births, age at first birth, HRT status, HRT use, age at menarche, BMI, height, physical activity, and alcohol intake
Itoh et al., (2014)	Case-Control	Japan	Diet	Postmenopausal	390	780	Highest (median:31.5) vs lowest (median:21.4) tertiles of cadmium intake (μg/day).	Age, residential area, moderate physical activity in the past 5 years, smoking status, family history of breast cancer, and number of births, isoflavone intake, vegetable intake, and total energy intake.
Nagata et al., (2013)	Case-Control	Japan	Biomarker (UCL)	Overall	153	584	Highest (>2.620) vs Lowest (1.674) tertile (μg/g creatinine)	Age, years of education, age at menarche, number of births, age at first birth, BMI, smoking status, alcohol intake, and family history of breast cancer among first-degree relatives
Adams et al., (2012)	Cohort	US	Diet	Postmenopausal	899	26,801	Highest (>13.3) vs. Lowest (<7.48) quartile (μg/day)	Age, total energy intake, education, race, HRT, vegetable consumption (excluding potatoes), potato consumption, whole grain consumption, cigarette smoking, BMI, physical activity, alcohol consumption, age at first childbirth, multivitamin use, and mammography.
Julin et al., (2012)	Cohort	Sweden	Diet	Postmenopausal	1916	55,987	Highest (>16) vs. Lowest (<13) tertiel (μg/day)	Age, BMI, >12 years of education, use of oral contraceptives, HRT, age at menarche, age at menopause, parity, age at first birth, alcohol consumption, glycemic load, and total energy intake, and intake of whole grain and vegetables in tertiles
Sawada et al., (2012)	Cohort	Japan	Diet	Overall	402	48,351	Highest (median:32.3) vs. Lowest (median:19.2) tertile (μg/day)	Age, area, BMI, smoking status, frequency of alcohol intake, leisure-time physical activity, intake of meat, soybean, vegetable, and fruit, menopausal status, and HRT
Gallagher et al., (2010)	Case-Control	US	Biomarker (UCL)	Overall	192	3174	Highest (≥0.60) vs. Lowest (<0.22) quartile (μg/g)	Age group, never-smoker, never-drinker, menopausal status, non-Hispanic white relative to black, Hispanic or Mexican American, multi-racial or other
McElroy et al., (2006)	Case-Control	US	Biomarker (UCL)	Overall	246	500	Highest (≥0.58 μg/g)) vs. Lower (<0.26) quartile (μg/g)	Age, parity, age at fi rst birth, family history of breast cancer, recent alcohol consumption, BMI, age at menarche, menopausal status, age at menopause, HRT, education, and marital status.

a From the most adjusted model. BMI: Body mass index; HRT: Hormone replacement therapy; BCL: Blood cadmium levels; UCL:Urinary cadmium levels.Articles are cited in descending order from the newest to the oldest.
