# Supplementary material for: Cadmium exposure and risk of breast cancer: A meta-analysis
Source: Environ Res. Author manuscript; Available in PMC 2023 Feb 24. (PMC9957608; doi:10.1016/j.envres.2022.115109)
Supplement: Multimedia component 1 [file NIHMS1861432-supplement-Multimedia_component_1.docx]

|  |  |  |
| --- | --- | --- |

**Supplemental Table S1**. Search strategy used in the review

| **Database** | **Included tittle** | **Results** |
| --- | --- | --- |
| **PubMed** | (("cadmium"[Title] OR "lead"[Title] OR "mercury"[Title] OR “chromium”[Title] OR “arsenic”[Title] OR "metallic air pollutants"[Title] OR "heavy metal"[Title]) AND ("breast cancer"[Title] OR "breast neoplasms"[Title] OR "breast tumor"[Title])) NOT ("review"[Title] OR "case report"[Title] OR "mice"[Title] OR "rat"[Title] OR "rats"[Title] OR "mouse"[Title] OR "in vitro"[Title]) | 2415 |
| **Scopus** | TITLE ( ( "cadmium" OR "lead" OR "arsenic" OR "chromium" OR "mercury" OR "metallic air pollutants" OR "heavy metal" ) AND ( "breast cancer" OR "breast neoplasms" OR "breast tumor" ) AND NOT ( "review" OR "case report" OR "mice" OR "rat" OR "rats" OR "mouse" OR "in vitro" ) ) | 249 |
| **Web of Science** | TITLE: ("cadmium" OR "lead" OR "arsenic" OR "chromium" OR "mercury" OR "metallic air pollutants" OR "heavy metal") AND TITLE: ("breast cancer" OR "breast neoplasms" OR "breast tumor") NOT TITLE: ("review"OR "case report" OR "mice" OR "rat" OR "rats" OR "mouse" OR "in vitro") | 91 |

**Supplemental Table S2**. Criteria adopted for Risk of Bias Assessment.

| **Domains** | **Level** | **Criteria** |
| --- | --- | --- |
| Bias due to confounding | Low | The study includes age, smoking habits, body mass index, hormone replacement therapy use, energy intake (only for dietary intake), and creatinine adjustment (only when the exposure measure was a urinary biomarker) as essential parts of the analysis plan |
|  | Moderate | The study includes age, smoking habits, body mass index and hormone replacement therapy use as essential parts of the analysis plan |
|  | High | The study includes age, smoking habits and body mass index as essential parts of the analysis plan |
| Bias in selecting participants in the study | Low | Selection of participants not related to cadmium exposure. |
|  | Moderate | Selection of participants is not clearly unrelated to cadmium exposure. |
|  | High | The selection of participants is not specified. |
| Bias in exposure classification | Low | Exposure is clearly classified using a biological sample for the assessment |
|  | Moderate | Possible exposure misclassification for studies using different records for the exposure assessment (e.g: air, medical records, math modeling, etc) or those using diet questionaries for evaluating the exposure in longitudinal fashion. |
|  | High | Studies use self-report for the exposure assessment or are based on an assessment performed after the beginning of the study (e.g: Case-control studies with exposure measurement based on diet questionaries). |
| Bias due to missing data | Low | If all variables used in analysis had < 10% missingness. |
|  | Moderate | If one or more variables used in analysis had 10% or more missingness but no variables had > 20% missingness. |
|  | High | If one or more variables used in analysis are 20% or more missing. |
| Bias in outcome measurement | Low | Breast cancer diagnosis used biopsy and clear description of technique with external validation. |
|  | Moderate | Breast cancer diagnosis based on health record review with or without external validation. |
|  | High | Breast cancer diagnosis was based on self-report only without external validation. |
| Bias in selection of reported results | Low | Evidence that results are clearly reported based on the prevalence/incidence of the disease as well as there are a clear report in inconsistencies about the sample size and final results. Also, there is a clear reporting of statistical methods and covariates information. In other words, the study results are fully reported, the authors included baseline and appropriated statistical methods. |
|  | Moderate | Evidence that results have not been a priory selected or biased. There is a good approach for reporting statistical methods, but it is unclear, or there is no description of how covariates were utilized. |
|  | High | There is evidence that results have been manipulated. There is no clear reporting of statistical methods. |
| Overall risk of bias | Low | If all domains were at low risk of bias, the overall risk was considered low. |
|  | Moderate | If at least one domain was found at moderate risk of bias, but without any high risk of bias rankings, the overall risk was considered moderate. |
|  | High | If at least one domain was found at high risk of bias, the overall risk was considered high. |

**Supplemental Table S3.** Summary Risk of Bias (RoB) assessment with overall study-level risk of bias.

| Author | Publication year | Bias due to confounding | Bias in selecting participants in the study | Bias in exposure classification | Bias due to missing data | Bias in outcome measurement | Bias in selection of reported results | **Study-level RoB Judgment** |
| --- | --- | --- | --- | --- | --- | --- | --- | --- |
| Andersson EM | 2021 | Low | Low | Low | Low | Moderate | Low | Moderate |
| Amadou A | 2020 | Low | Low | Moderate | Moderate | Moderate | Low | Moderate |
| White AJ | 2019 | Moderate | Low | Moderate | Low | Moderate | Low | Moderate |
| Gaudet MM | 2019 | Low | Low | Low | Low | Moderate | Low | Moderate |
| Grioni S | 2019 | Low | Low | Low | Low | Moderate | Low | Moderate |
| Strumylaite L | 2019 | Low | Low | Low | Low | Moderate | Low | Moderate |
| Adams SV | 2016 | Low | Low | Low | Low | Moderate | Low | Moderate |
| Eriksen KT | 2016 | Low | Low | Low | Low | Moderate | Low | Moderate |
| Wei XL | 2015 | Low | Low | Low | Low | Moderate | Low | Moderate |
| Adams SV | 2014 | Low | Low | Moderate | Low | Moderate | Low | Moderate |
| Eriksen KT | 2014 | Moderate | Low | Moderate | Low | Moderate | Low | Moderate |
| Itoh H | 2014 | Low | Low | Moderate | Low | Moderate | Low | Moderate |
| Nagata C | 2013 | Moderate | Low | Low | Low | Moderate | Low | Moderate |
| Adams SV | 2012 | Low | Low | Moderate | Low | Moderate | Low | Moderate |
| Julin B | 2012 | Moderate | Low | Moderate | Low | Moderate | Low | Moderate |
| Sawada | 2012 | Low | Low | Moderate | Low | Moderate | Low | Moderate |
| Gallagher CM | 2010 | High | Low | Low | Low | High | Low | High |
| McElroy JA | 2006 | Low | Low | Moderate | Low | Moderate | Low | Moderate |

**Supplemental Figure S1.** Forest plot of cadmium in biomarkers and female breast cancer.

**a) Overall**

**
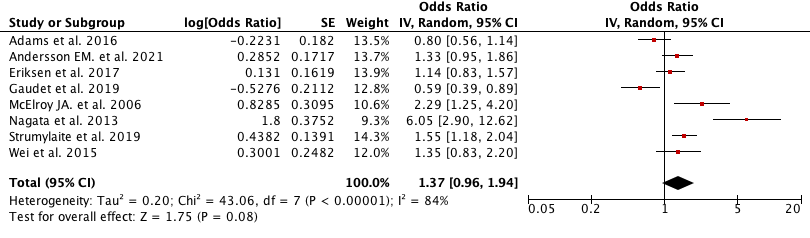
**

**b) Postmenopausal**

**
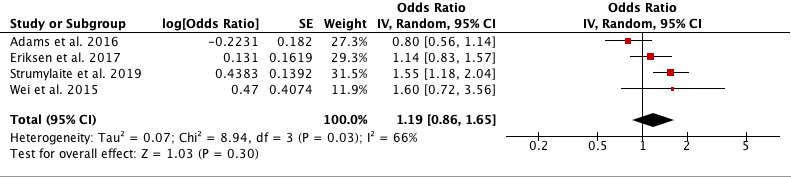
**

**Supplemental Figure S2.** Forest plot dietary cadmium and female breast cancer

**a) Overall**

**
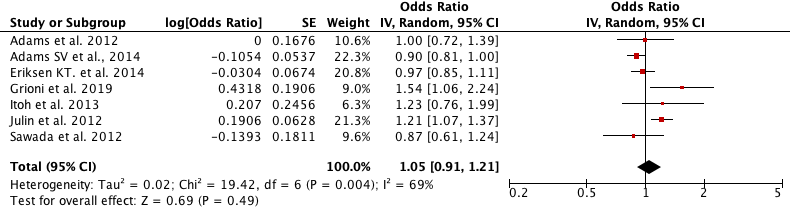
**

**b) Premenopausal**

**
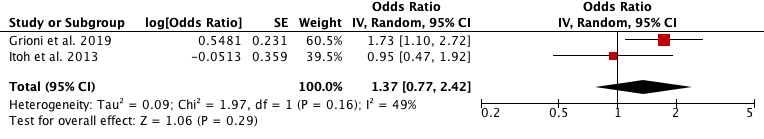
**

**c) Postmenopausal** **
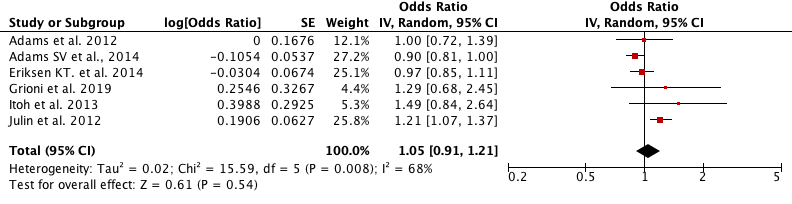
**

**Supplemental Figure S3.** Forest plot airborne cadmium and female breast cancer.

**a) Overall women**

**
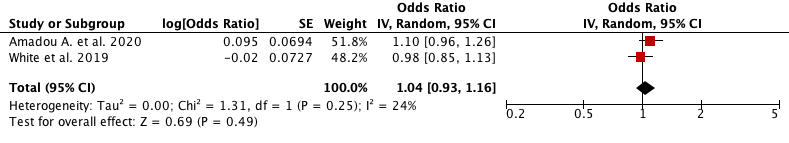
**

**b) Premenopausal**

**
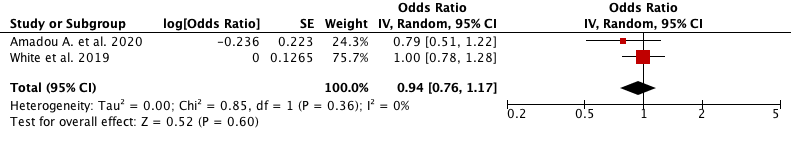
**

**d) Postmenopausal**

**
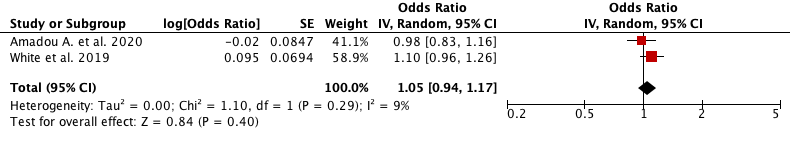
**

**Supplemental Table S4.** PRISMA checklist.

| **Section and Topic** | **Item #** | **Checklist item** | **Location where item is reported** |
| --- | --- | --- | --- |
| **TITLE** | | | Page |
| Title | 1 | Identify the report as a systematic review. | 1 |
| **ABSTRACT** | | |  |
| Abstract | 2 | See the PRISMA 2020 for Abstracts checklist. | 2 |
| **INTRODUCTION** | | |  |
| Rationale | 3 | Describe the rationale for the review in the context of existing knowledge. | 3-4 |
| Objectives | 4 | Provide an explicit statement of the objective(s) or question(s) the review addresses. | 5 |
| **METHODS** | | |  |
| Eligibility criteria | 5 | Specify the inclusion and exclusion criteria for the review and how studies were grouped for the syntheses. | 5 |
| Information sources | 6 | Specify all databases, registers, websites, organisations, reference lists and other sources searched or consulted to identify studies. Specify the date when each source was last searched or consulted. | 6-7 |
| Search strategy | 7 | Present the full search strategies for all databases, registers and websites, including any filters and limits used. | 7 |
| Selection process | 8 | Specify the methods used to decide whether a study met the inclusion criteria of the review, including how many reviewers screened each record and each report retrieved, whether they worked independently, and if applicable, details of automation tools used in the process. | 7-8 |
| Data collection process | 9 | Specify the methods used to collect data from reports, including how many reviewers collected data from each report, whether they worked independently, any processes for obtaining or confirming data from study investigators, and if applicable, details of automation tools used in the process. | 8 |
| Data items | 10a | List and define all outcomes for which data were sought. Specify whether all results that were compatible with each outcome domain in each study were sought (e.g. for all measures, time points, analyses), and if not, the methods used to decide which results to collect. | 8 |
|  | 10b | List and define all other variables for which data were sought (e.g. participant and intervention characteristics, funding sources). Describe any assumptions made about any missing or unclear information. | 8 |
| Study risk of bias assessment | 11 | Specify the methods used to assess risk of bias in the included studies, including details of the tool(s) used, how many reviewers assessed each study and whether they worked independently, and if applicable, details of automation tools used in the process. | 8 |
| Effect measures | 12 | Specify for each outcome the effect measure(s) (e.g. risk ratio, mean difference) used in the synthesis or presentation of results. | 8 |
| Synthesis methods | 13a | Describe the processes used to decide which studies were eligible for each synthesis (e.g. tabulating the study intervention characteristics and comparing against the planned groups for each synthesis (item #5)). | 9 |
|  | 13b | Describe any methods required to prepare the data for presentation or synthesis, such as handling of missing summary statistics, or data conversions. | 9 |
|  | 13c | Describe any methods used to tabulate or visually display results of individual studies and syntheses. | 9 |
|  | 13d | Describe any methods used to synthesize results and provide a rationale for the choice(s). If meta-analysis was performed, describe the model(s), method(s) to identify the presence and extent of statistical heterogeneity, and software package(s) used. | 9 |
|  | 13e | Describe any methods used to explore possible causes of heterogeneity among study results (e.g. subgroup analysis, meta-regression). | 9 |
|  | 13f | Describe any sensitivity analyses conducted to assess robustness of the synthesized results. | - |
| Reporting bias assessment | 14 | Describe any methods used to assess risk of bias due to missing results in a synthesis (arising from reporting biases). | Supplemental tables S3 and S3 |
| Certainty assessment | 15 | Describe any methods used to assess certainty (or confidence) in the body of evidence for an outcome. | - |
| **RESULTS** | | |  |
| Study selection | 16a | Describe the results of the search and selection process, from the number of records identified in the search to the number of studies included in the review, ideally using a flow diagram. | 9-10 |
|  | 16b | Cite studies that might appear to meet the inclusion criteria, but which were excluded, and explain why they were excluded. | 10 |
| Study characteristics | 17 | Cite each included study and present its characteristics. | 10 |
| Risk of bias in studies | 18 | Present assessments of risk of bias for each included study. | Supplemental tables S3 and S3 |
| Results of individual studies | 19 | For all outcomes, present, for each study: (a) summary statistics for each group (where appropriate) and (b) an effect estimate and its precision (e.g. confidence/credible interval), ideally using structured tables or plots. | 10-11 |
| Results of syntheses | 20a | For each synthesis, briefly summarise the characteristics and risk of bias among contributing studies. | 11 |
|  | 20b | Present results of all statistical syntheses conducted. If meta-analysis was done, present for each the summary estimate and its precision (e.g. confidence/credible interval) and measures of statistical heterogeneity. If comparing groups, describe the direction of the effect. | 11 |
|  | 20c | Present results of all investigations of possible causes of heterogeneity among study results. | 11 |
|  | 20d | Present results of all sensitivity analyses conducted to assess the robustness of the synthesized results. | 11 |
| Reporting biases | 21 | Present assessments of risk of bias due to missing results (arising from reporting biases) for each synthesis assessed. | 10 |
| Certainty of evidence | 22 | Present assessments of certainty (or confidence) in the body of evidence for each outcome assessed. | - |
| **DISCUSSION** | | |  |
| Discussion | 23a | Provide a general interpretation of the results in the context of other evidence. | 11-14 |
|  | 23b | Discuss any limitations of the evidence included in the review. | 15-16 |
|  | 23c | Discuss any limitations of the review processes used. | 15-16 |
|  | 23d | Discuss implications of the results for practice, policy, and future research. | 15-16 |
| **OTHER INFORMATION** | | |  |
| Registration and protocol | 24a | Provide registration information for the review, including register name and registration number, or state that the review was not registered. | http://www.crd.york.ac.uk/PROSPERO/; CRD42022341929 |
|  | 24b | Indicate where the review protocol can be accessed, or state that a protocol was not prepared. | - |
|  | 24c | Describe and explain any amendments to information provided at registration or in the protocol. | - |
| Support | 25 | Describe sources of financial or non-financial support for the review, and the role of the funders or sponsors in the review. | No financial support |
| Competing interests | 26 | Declare any competing interests of review authors. | No competing interest |
| Availability of data, code and other materials | 27 | Report which of the following are publicly available and where they can be found: template data collection forms; data extracted from included studies; data used for all analyses; analytic code; any other materials used in the review. | - |

*From:*  Page MJ, McKenzie JE, Bossuyt PM, Boutron I, Hoffmann TC, Mulrow CD, et al. The PRISMA 2020 statement: an updated guideline for reporting systematic reviews. BMJ 2021;372:n71. doi: 10.1136/bmj.n71
